# A commentary on ‘The value of cholinesterase inhibitors for improving neuropsychiatric and functional assessment scores in patients with Alzheimer disease: a systematic review and meta-analysis of on placebo-controlled RCTs’

**DOI:** 10.1097/JS9.0000000000001548

**Published:** 2024-05-03

**Authors:** Yawen He, Zhiyang Jiang, Yonghua Zhang

**Affiliations:** Hangzhou TCM Hospital of Zhejiang Chinese Medical University, Hangzhou, Zhejiang, People’s Republic of China

*Dear Editor,*

We are interested in the article entitled ʻThe value of cholinesterase inhibitors for improving neuropsychiatric and functional assessment scores in patients with Alzheimer disease: a systematic review and meta-analysis of on placebo-controlled RCTs,ʼ which was published in the *International Journal of Surgery* ^1^. The meta-analysis included 12 randomized, double-blind, placebo-controlled trials with 6908 participants. Their findings suggest that cholinesterase inhibitors (ChEIs) treatment generally improved neuropsychiatric and functional assessment scores in patients with Alzheimer disease (AD). These findings are both timely and of great significance. However, this article found the intolerance of ChEIs always focused on the gastrointestinal (GI) system, with significant differences in adverse events (AEs) between ChEIs and placebo, which could lead to treatment discontinuation. It indicated that ChEIs indicated the incidence of GI system disorders [odds ratio (OR) 1.78; 95% CI 1.44–2.21], anorexia (OR 2.14; 95% CI 1.62–2.83), nausea (OR 2.86; 95% CI 2.32–3.52), diarrhea (OR 1.46; 95% CI 1.16–1.83), vomiting (OR 2.54; 95% CI 1.97–3.28), and constipation (OR 2.48; 95% CI 1.43–4.30).

Transdermal delivery of donepezil bypasses the digestive tract, which can reduce the possibility of side effects, including GI AEs^2^. In addition, this route maintains steady concentrations of the drug within the therapeutic range, often without the need for daily dosing, which is particularly important for patients with AD, as those with dementia may forget to take or resist taking their medication. It is also helpful in improving patient compliance.

In 2022, a donepezil transdermal delivery system (TDS) designed to deliver donepezil through the skin during a 7-day wear period was approved by the US FDA^3^. This TDS is available in 5-mg/day and 10-mg/day dosages. Results of a previous study showed that 10-mg/day and dose-normalized 5-mg/day donepezil TDS are bioequivalent to 10-mg/day of oral donepezil^4^. GI disorders (constipation, nausea, diarrhea, and vomiting) were more frequent when participants were taking oral donepezil than when they were using donepezil TDS.

Although some people are worried about skin irritation caused by TDS, a randomized, double-blind study indicates that the donepezil TDS under investigation was generally well-tolerated locally with none to mild skin irritation under exaggerated conditions of use (3 consecutive weekly patch applications to the same skin site)^5^.

Again, we thank Zhang *et al*. for their meaningful work in summarizing the evidence of ChEIs in the treatment of AD. We made a preliminary reflection on the meaningful questions they raised. It is hoped that there will be more studies in the future that can provide a clinical reference in this regard.

## Ethical approval

Not applicable.

## Consent

Not applicable.

## Sources of funding

Not applicable.

## Author contribution

Y.H., Z.J., and Y.Z.: conception and design of the study; Y.H. and Z.J.: collected and analyzed the data and also wrote the paper. All the authors drafted and revised the paper. Additionally, all authors have read and approved the final manuscript.

## Conflicts of interest disclosure

The authors declare no conflicts of interes.

## Research registration unique identifying number (UIN)

Not applicable.

## Guarantor

Yonghua Zhang accepts full responsibility for the work and the conduct of the study.

## Data availability statement

This manuscript is a comment. Don’t need a data availability statement. However, all the data from the current study are publicly available.

## Provenance and peer review

Not applicable.

## References

[1] Zhang Y Sun Y Hu X . The value of cholinesterase inhibitors for improving neuropsychiatric and functional assessment scores in patients with Alzheimer disease: a systematic review and meta-analysis of on placebo-controlled RCTs. Int J Surg 2024;3. [Epub ahead of print]. doi:10.1097/JS9.0000000000001381 PMC11175821 38573101

[2] Paudel KS Milewski M Swadley CL . Challenges and opportunities in dermal/transdermal delivery. Ther Deliv 2010;1:109–131. 21132122 10.4155/tde.10.16 PMC2995530

[3] ADLARITY (donepezil transdermal system), Prescribing information, 2022. Accessed 3 April 2023.

[4] Tariot PN Braeckman R Oh C . Comparison of steady-state pharmacokinetics of donepezil transdermal delivery system with oral donepezil. J Alzheimers Dis 2022;90:161–172. 36120781 10.3233/JAD-220530 PMC9661317

[5] Sabbagh MN Mathew P Blau A . A randomized double-blind study to assess the skin irritation and sensitization potential of a once-weekly donepezil transdermal delivery system in healthy volunteers. Alzheimer Dis Assoc Disord 2023;37:290–295. 37695107 10.1097/WAD.0000000000000578 PMC10664792

